# Methyl *N*-(4-nitro­phen­yl)carbamate

**DOI:** 10.1107/S1600536811018757

**Published:** 2011-05-25

**Authors:** Qun Cai, Zhuan Fei, Lin Li

**Affiliations:** a Key Laboratory of Pesticide and Chemical Biology of the Ministry of Education, College of Chemistry, Central China Normal University, Wuhan 430079, People’s Republic of China

## Abstract

In the title mol­ecule, C_8_H_8_N_2_O_4_, the nitro and meth­oxy­carbonyl groups are twisted from the plane of aromatic ring by 5.1 (1) and 6.2 (1)°, respectively. In the crystal, inter­molecular N—H⋯O hydrogen bonds link the mol­ecules related by translation along the *b* axis into chains. Weak inter­molecular C—H⋯O inter­actions link further these chains into sheets parallel to the *bc* plane.

## Related literature

For the preparation of the title compound, see: Wilshire (1990 ▶). For a related structure, see: Yakimanski *et al.* (1997 ▶).
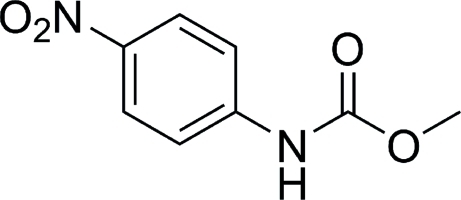


## Experimental

### 

#### Crystal data

C_8_H_8_N_2_O_4_
*M* *_r_* = 196.16
Triclinic, 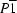

*a* = 7.4269 (11) Å
*b* = 8.1003 (12) Å
*c* = 8.5376 (12) Å
α = 101.634 (2)°
β = 97.914 (2)°
γ = 116.660 (2)°
*V* = 434.04 (11) Å^3^
*Z* = 2
Mo *K*α radiation
μ = 0.12 mm^−1^
*T* = 298 K
0.40 × 0.30 × 0.04 mm

#### Data collection

Bruker SMART APEX diffractometer
4507 measured reflections
1686 independent reflections
1539 reflections with *I* > 2σ(*I*)
*R*_int_ = 0.051

#### Refinement

*R*[*F* ^2^ > 2σ(*F* ^2^)] = 0.074
*wR*(*F* ^2^) = 0.162
*S* = 1.26
1686 reflections
132 parameters
H atoms treated by a mixture of independent and constrained refinement
Δρ_max_ = 0.21 e Å^−3^
Δρ_min_ = −0.32 e Å^−3^

### 

Data collection: *SMART* (Bruker, 1997 ▶); cell refinement: *SAINT* (Bruker, 1999 ▶); data reduction: *SAINT*; program(s) used to solve structure: *SHELXS97* (Sheldrick, 2008 ▶); program(s) used to refine structure: *SHELXL97* (Sheldrick, 2008 ▶); molecular graphics: *SHELXTL* (Sheldrick, 2008 ▶); software used to prepare material for publication: *SHELXL97*.

## Supplementary Material

Crystal structure: contains datablocks I, global. DOI: 10.1107/S1600536811018757/cv5074sup1.cif

Structure factors: contains datablocks I. DOI: 10.1107/S1600536811018757/cv5074Isup2.hkl

Supplementary material file. DOI: 10.1107/S1600536811018757/cv5074Isup3.cml

Additional supplementary materials:  crystallographic information; 3D view; checkCIF report

## Figures and Tables

**Table 1:** Hydrogen-bond geometry (Å, °)

*D*—H⋯*A*	*D*—H	H⋯*A*	*D*⋯*A*	*D*—H⋯*A*
C7—H7⋯O2^i^	0.93	2.57	3.471 (3)	163
C1—H1*B*⋯O4^ii^	0.96	2.53	3.324 (4)	140
N1—H1⋯O3^iii^	0.82 (3)	2.20 (4)	3.016 (3)	170 (3)

Symmetry codes: (i) 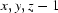; (ii) 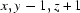; (iii) 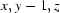.

**Fig. 1.**
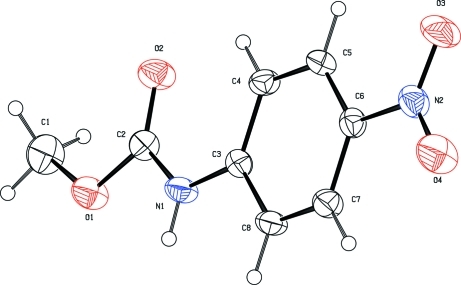
A view of (I), showing the atom-labelling scheme, with displacement ellipsoids drawn at the 30% probability level.

## supplementary crystallographic information

### Comment

4-Nitrophenylhexyl derivatives are studied in the crystal engineering and design of nonlinear optical (NLO) materials (Yakimanski et al., 1997). Herewith we report the crystal structure of the title compound (I).

In (I) (Fig. 1), all bond lengths and angles are normal and comparable with those observed in 4-nitrophenyl-hexyl-urethane (Yakimanski et al., 1997). The nitro and methoxycarbonyl groups are twisted from the plane of aromatic ring at 5.1 (1) and 6.2 (1)°, respectively. In the crystal structure, intermolecular N—H···O hydrogen bonds (Table 1) link the molecules related by translation along axis b<ι> into chains. Weak intermolecular C—H···O interactions (Table 1) link further these chains into sheets parallel to bc<ι> plane.

### Experimental

The title compound was synthesized according to Wilshire (1990). Crystals of (I) suitable for X-ray diffraction were grown by slow evaporation of a chloroform-methanol (2:1) solution of the title compound under 293 K.

### Refinement

C-bound H atoms were positioned in geometrically idealized positions and constrained to ride on their parent atoms, with C—H distances in the range 0.93–0.98 Å and *U*_iso_(H) = 1.2*U*_eq_(C) or *U*_iso_(H) = 1.5*U*_eq_(C). Atom H1 was located on difference map and isotropically refined.

### Crystal data

**Table:**

C_8_H_8_N_2_O_4_	*Z* = 2
*M**_r_* = 196.16	*F*(000) = 204
Triclinic, *P*1	*D*_x_ = 1.501 Mg m^−^^3^
Hall symbol: -P 1	Mo *K*α radiation, λ = 0.71073 Å
*a* = 7.4269 (11) Å	Cell parameters from 2193 reflections
*b* = 8.1003 (12) Å	θ = 2.5–28.2°
*c* = 8.5376 (12) Å	µ = 0.12 mm^−^^1^
α = 101.634 (2)°	*T* = 298 K
β = 97.914 (2)°	Block, yellow
γ = 116.660 (2)°	0.40 × 0.30 × 0.04 mm
*V* = 434.04 (11) Å^3^	

### Data collection

**Table:**

Bruker SMART APEX diffractometer	1539 reflections with *I* > 2σ(*I*)
Radiation source: fine-focus sealed tube	*R*_int_ = 0.051
graphite	θ_max_ = 26.0°, θ_min_ = 2.5°
φ and ω scans	*h* = −9→9
4507 measured reflections	*k* = −9→9
1686 independent reflections	*l* = −10→10

### Refinement

**Table:**

Refinement on *F*^2^	Primary atom site location: structure-invariant direct methods
Least-squares matrix: full	Secondary atom site location: difference Fourier map
*R*[*F*^2^ > 2σ(*F*^2^)] = 0.074	Hydrogen site location: inferred from neighbouring sites
*wR*(*F*^2^) = 0.162	H atoms treated by a mixture of independent and constrained refinement
*S* = 1.26	*w* = 1/[σ^2^(*F*_o_^2^) + (0.047*P*)^2^ + 0.2842*P*] where *P* = (*F*_o_^2^ + 2*F*_c_^2^)/3
1686 reflections	(Δ/σ)_max_ < 0.001
132 parameters	Δρ_max_ = 0.21 e Å^−^^3^
0 restraints	Δρ_min_ = −0.32 e Å^−^^3^

### Special details

**Table:**

Geometry. All e.s.d.'s (except the e.s.d. in the dihedral angle between two l.s. planes) are estimated using the full covariance matrix. The cell e.s.d.'s are taken into account individually in the estimation of e.s.d.'s in distances, angles and torsion angles; correlations between e.s.d.'s in cell parameters are only used when they are defined by crystal symmetry. An approximate (isotropic) treatment of cell e.s.d.'s is used for estimating e.s.d.'s involving l.s. planes.
Refinement. Refinement of *F*^2^ against ALL reflections. The weighted *R*-factor *wR* and goodness of fit *S* are based on *F*^2^, conventional *R*-factors *R* are based on *F*, with *F* set to zero for negative *F*^2^. The threshold expression of *F*^2^ > σ(*F*^2^) is used only for calculating *R*-factors(gt) *etc*. and is not relevant to the choice of reflections for refinement. *R*-factors based on *F*^2^ are statistically about twice as large as those based on *F*, and *R*- factors based on ALL data will be even larger.

### Fractional atomic coordinates and isotropic or equivalent isotropic displacement parameters (Å^2^)

**Table:**

	*x*	*y*	*z*	*U*_iso_*/*U*_eq_	
C1	0.2798 (6)	0.1171 (5)	0.4095 (4)	0.0645 (10)	
H1A	0.4024	0.2283	0.4846	0.097*	
H1B	0.2871	0.0023	0.4122	0.097*	
H1C	0.1582	0.1095	0.4419	0.097*	
C2	0.2591 (4)	0.2904 (4)	0.2229 (3)	0.0400 (6)	
C3	0.2433 (4)	0.4234 (4)	−0.0116 (3)	0.0351 (6)	
C4	0.2670 (4)	0.5997 (4)	0.0750 (3)	0.0391 (6)	
H4	0.2853	0.6318	0.1891	0.047*	
C5	0.2633 (4)	0.7260 (4)	−0.0091 (3)	0.0399 (6)	
H5	0.2796	0.8445	0.0481	0.048*	
C6	0.2355 (4)	0.6774 (4)	−0.1782 (3)	0.0377 (6)	
C7	0.2141 (5)	0.5042 (4)	−0.2666 (3)	0.0427 (7)	
H7	0.1979	0.4739	−0.3804	0.051*	
C8	0.2173 (4)	0.3786 (4)	−0.1826 (3)	0.0415 (7)	
H8	0.2018	0.2607	−0.2405	0.050*	
N2	0.2288 (4)	0.8111 (4)	−0.2670 (3)	0.0479 (6)	
N1	0.2450 (4)	0.2846 (4)	0.0624 (3)	0.0436 (6)	
O1	0.2673 (4)	0.1340 (3)	0.2437 (2)	0.0538 (6)	
O2	0.2635 (4)	0.4136 (3)	0.3310 (2)	0.0545 (6)	
O3	0.2327 (4)	0.9584 (3)	−0.1911 (3)	0.0695 (7)	
O4	0.2176 (4)	0.7718 (4)	−0.4139 (3)	0.0733 (8)	
H1	0.235 (5)	0.187 (5)	0.001 (4)	0.059 (10)*	

### Atomic displacement parameters (Å^2^)

**Table:**

	*U*^11^	*U*^22^	*U*^33^	*U*^12^	*U*^13^	*U*^23^
C1	0.089 (3)	0.073 (2)	0.0476 (18)	0.045 (2)	0.0229 (17)	0.0351 (17)
C2	0.0426 (16)	0.0416 (16)	0.0391 (14)	0.0211 (13)	0.0138 (12)	0.0152 (13)
C3	0.0356 (14)	0.0342 (14)	0.0388 (14)	0.0188 (12)	0.0108 (11)	0.0128 (11)
C4	0.0481 (16)	0.0400 (15)	0.0286 (12)	0.0224 (13)	0.0106 (11)	0.0064 (11)
C5	0.0458 (16)	0.0310 (14)	0.0441 (15)	0.0224 (13)	0.0101 (12)	0.0061 (11)
C6	0.0362 (14)	0.0361 (15)	0.0429 (14)	0.0185 (12)	0.0089 (11)	0.0150 (12)
C7	0.0567 (18)	0.0455 (17)	0.0338 (14)	0.0307 (15)	0.0129 (12)	0.0129 (12)
C8	0.0562 (18)	0.0350 (15)	0.0365 (14)	0.0271 (14)	0.0122 (12)	0.0051 (11)
N2	0.0539 (15)	0.0430 (15)	0.0545 (15)	0.0282 (13)	0.0137 (12)	0.0194 (12)
N1	0.0675 (17)	0.0398 (14)	0.0324 (12)	0.0334 (13)	0.0155 (11)	0.0101 (10)
O1	0.0864 (16)	0.0512 (13)	0.0429 (11)	0.0426 (12)	0.0242 (11)	0.0254 (10)
O2	0.0838 (16)	0.0558 (13)	0.0366 (11)	0.0421 (12)	0.0224 (10)	0.0157 (10)
O3	0.106 (2)	0.0434 (13)	0.0709 (16)	0.0476 (14)	0.0179 (14)	0.0174 (11)
O4	0.120 (2)	0.0758 (17)	0.0552 (15)	0.0635 (17)	0.0334 (14)	0.0378 (13)

### Geometric parameters (Å, °)

**Table:**

C1—O1	1.444 (3)	C4—H4	0.9300
C1—H1A	0.9600	C5—C6	1.378 (4)
C1—H1B	0.9600	C5—H5	0.9300
C1—H1C	0.9600	C6—C7	1.379 (4)
C2—O2	1.200 (3)	C6—N2	1.456 (3)
C2—O1	1.340 (3)	C7—C8	1.364 (4)
C2—N1	1.350 (3)	C7—H7	0.9300
C3—C4	1.390 (4)	C8—H8	0.9300
C3—C8	1.394 (4)	N2—O4	1.211 (3)
C3—N1	1.400 (3)	N2—O3	1.223 (3)
C4—C5	1.370 (4)	N1—H1	0.82 (3)
			
O1—C1—H1A	109.5	C6—C5—H5	120.0
O1—C1—H1B	109.5	C5—C6—C7	121.6 (2)
H1A—C1—H1B	109.5	C5—C6—N2	119.7 (2)
O1—C1—H1C	109.5	C7—C6—N2	118.7 (2)
H1A—C1—H1C	109.5	C8—C7—C6	118.3 (2)
H1B—C1—H1C	109.5	C8—C7—H7	120.9
O2—C2—O1	124.7 (3)	C6—C7—H7	120.9
O2—C2—N1	126.5 (3)	C7—C8—C3	121.3 (2)
O1—C2—N1	108.8 (2)	C7—C8—H8	119.3
C4—C3—C8	119.3 (2)	C3—C8—H8	119.3
C4—C3—N1	124.0 (2)	O4—N2—O3	122.3 (3)
C8—C3—N1	116.7 (2)	O4—N2—C6	118.9 (2)
C5—C4—C3	119.5 (2)	O3—N2—C6	118.9 (2)
C5—C4—H4	120.3	C2—N1—C3	127.9 (2)
C3—C4—H4	120.3	C2—N1—H1	116 (2)
C4—C5—C6	120.0 (2)	C3—N1—H1	116 (2)
C4—C5—H5	120.0	C2—O1—C1	115.7 (2)
			
C8—C3—C4—C5	0.4 (4)	C5—C6—N2—O4	175.4 (3)
N1—C3—C4—C5	179.9 (3)	C7—C6—N2—O4	−4.5 (4)
C3—C4—C5—C6	0.2 (4)	C5—C6—N2—O3	−5.3 (4)
C4—C5—C6—C7	−1.0 (4)	C7—C6—N2—O3	174.8 (3)
C4—C5—C6—N2	179.2 (2)	O2—C2—N1—C3	3.5 (5)
C5—C6—C7—C8	1.2 (4)	O1—C2—N1—C3	−176.5 (3)
N2—C6—C7—C8	−179.0 (2)	C4—C3—N1—C2	3.4 (5)
C6—C7—C8—C3	−0.6 (4)	C8—C3—N1—C2	−177.0 (3)
C4—C3—C8—C7	−0.2 (4)	O2—C2—O1—C1	0.8 (4)
N1—C3—C8—C7	−179.8 (3)	N1—C2—O1—C1	−179.2 (3)

### Hydrogen-bond geometry (Å, °)

**Table:**

*D*—H···*A*	*D*—H	H···*A*	*D*···*A*	*D*—H···*A*
C7—H7···O2^i^	0.93	2.57	3.471 (3)	163
C4—H4···O2	0.93	2.30	2.892 (3)	121
C1—H1B···O4^ii^	0.96	2.53	3.324 (4)	140
N1—H1···O3^iii^	0.82 (3)	2.20 (4)	3.016 (3)	170 (3)

Symmetry codes: (i) *x*, *y*, *z*−1; (ii) *x*, *y*−1, *z*+1; (iii) *x*, *y*−1, *z*.
